# Nitric Oxide Modulates Postnatal Bone Marrow-Derived Mesenchymal Stem Cell Migration

**DOI:** 10.3389/fcell.2016.00133

**Published:** 2016-11-24

**Authors:** John W. Fuseler, Mani T. Valarmathi

**Affiliations:** ^1^Department of Pathology, Microbiology and Immunology, School of Medicine, University of South CarolinaColumbia, SC, USA; ^2^Department of Comparative Biosciences, College of Veterinary Medicine, University of Illinois at Urbana-ChampaignUrbana, IL, USA

**Keywords:** mesenchymal stem cells, bone marrow stromal cells, adult stem cell migration, adult stem cell proliferation, nitric oxide, fractal analysis, cytoskeleton, actin

## Abstract

Nitric oxide (NO) is a small free-radical gas molecule, which is highly diffusible and can activate a wide range of downstream effectors, with rapid and widespread cellular effects. NO is a versatile signaling mediator with a plethora of cellular functions. For example, NO has been shown to regulate actin, the microfilament, dependent cellular functions, and also acts as a putative stem cell differentiation-inducing agent. In this study, using a wound-healing model of cellular migration, we have explored the effect of exogenous NO on the kinetics of movement and morphological changes in postnatal bone marrow-derived mesenchymal stem cells (MSCs). Cellular migration kinetics and morphological changes of the migrating MSCs were measured in the presence of an NO donor (S-Nitroso-N-Acetyl-D,L-Penicillamine, SNAP), especially, to track the dynamics of single-cell responses. Two experimental conditions were assessed, in which SNAP (200 μM) was applied to the MSCs. In the first experimental group (SN-1), SNAP was applied immediately following wound formation, and migration kinetics were determined for 24 h. In the second experimental group (SN-2), MSCs were pretreated for 7 days with SNAP prior to wound formation and the determination of migration kinetics. The generated displacement curves were further analyzed by non-linear regression analysis. The migration displacement of the controls and NO treated MSCs (SN-1 and SN-2) was best described by a two parameter exponential functions expressing difference constant coefficients. Additionally, changes in the fractal dimension (*D*) of migrating MSCs were correlated with their displacement kinetics for all the three groups. Overall, these data suggest that NO may evidently function as a stop migration signal by disordering the cytoskeletal elements required for cell movement and proliferation of MSCs.

## Introduction

Nitric oxide (NO) is a small free-radical gas molecule that can easily traverse cell membranes by virtue of its small size and uncharged nature, and is involved in a wide range of biological events in almost all living systems. The regulation and modification of NO production are the key steps that govern various biological processes, both in physiological and pathophysiological conditions. NO plays a crucial role in smooth muscle relaxation and vessel dilatation, thereby regulating blood flow and blood pressure (Cohen, 1995; Umans and Levi, 1995). Beyond its vascular functions, NO has been demonstrated to regulate numerous other cellular functions, for example, functions that are associated with actin and microtubule cytoskeletal system, including orchestrated cellular movement and migration (Sarkar et al., 1996; Kiviluoto et al., 2001; Seymour et al., 2010); as well as cellular proliferation and cell division, including mitosis and meiosis (Ignarro et al., 2001; Chen et al., 2004; Laguinge et al., 2004; Pandey et al., 2010). These functions are also dependent on various degrees of cytoskeletal organization and functionally links NO to cytoskeletal function and integrity (Fuseler and Valarmathi, 2012). NO is also involved in embryonic development and its associated migration of stem and progenitor cells in various organs and organ systems, especially the heart (Lee et al., 2000; Feng et al., 2002). Nitric oxide and the nitric oxide synthase isoforms (NOS) have been demonstrated to facilitate the differentiation process of numerous cell types that include nerve cells, various tumor cells, and embryonic stem cells into cells resembling those of myocardial lineage (Jenkins et al., 1995; Peunova and Enikolopov, 1995; Jadeski et al., 2003; Kanno et al., 2004; Mujoo et al., 2008; Bian and Murad, 2014; Hodge et al., 2016). Thus, NO is a versatile signaling mediator with a plethora of cellular functions.

With respect to cellular migration and proliferation, NO expresses its classical Janus Effect, i.e., depending upon the conditions, tissue locations, and cell types, NO either stimulates or suppresses cellular movement and/or proliferation. NO has been shown to be a requirement or a stimulus for cellular migration and/or proliferation of endothelial cells. When umbilical vein endothelial cells (HUVECs) were exposed to ischemia/reperfusion (I/R) injury, the iNOS dependent NO, enhanced cellular migration and apoptosis; whereas, the iNOS specific inhibitor, L-NAME, suppressed cellular migration and movement of these cells (Zhu et al., 2016). In endothelial cells, NO has been further implicated in promoting cellular migration by the indirect activation of Rac-1 and changes in cytoskeletal morphology that were associated with cell movement (Eller-Borges et al., 2015). Additionally, NO derived from eNOS, has been shown to mediate vascular endothelial growth factor (VEGF) mediated stimulation of cytoskeletal remodeling and cellular migration in endothelial cells. The novel link in this pathway of activation was proposed to be the S-nitrosylation of the small actin binding protein, cofilin-1 (CFL-1), which was essential for actin cytoskeleton remodeling and subsequent cellular movement (Zhang et al., 2015). NO expression has also been demonstrated to increase the migration and proliferation of other cell types of diverse lineages. In the microglial cell line (BV2) and primary microglia, NO donors increased cellular migration and movement in a classical scratch-wound assay, whereas, NO scavengers reduced their motility (Scheiblich et al., 2014). Prostate cancer cells (cell lines PC3 and DU145) in response to a photodynamic therapy-like challenge that enhanced the NO expression resulted in an increase in the migration rates, proliferation, and invasiveness of these cell types. These effects were attenuated by inhibition of iNOS as well as the suppression of NO (Fahey and Girotti, 2015). These observations suggested that increases in NO level may incur potentially tumor promoting side effects under certain conditions in the neoplastic cells. These and related studies demonstrated a role for NO as a positive regulator of cellular migration and proliferation in both normal and neoplastic cells (Duan et al., 2009; Lu et al., 2015; Yongsanguanchai et al., 2015).

Conversely, the presence of NO has been shown to suppress cellular migration and proliferation in various cell types. In HepG2 tumor cells, the presence of NO significantly inhibited cellular proliferation. Importantly, NO effectively inhibited the ability of migration and invasiveness of these tumor cells. The degree of inhibition of proliferation and migration capacity was directly proportional to the concentration of the NO donor (Zhou et al., 2016). Similar dose dependent inhibitory results on cellular proliferation have been observed in gastric cancer cells that were treated with an NO donor (Sang et al., 2011). Expression of NO was also observed to block cellular migration of HeLa cells and endothelial cells by blunting the phosphorylation of Akt/PKB (Bulotta et al., 2009). The elevation of NO resulted in G0-G1 arrest, suppressing mitosis and cellular proliferation. Elevated NO also inhibited gastric cancer cell growth by modulation of the Akt pathway (Sang et al., 2011). Increased NO has also been demonstrated to suppress the capacity for migration and survival of cutaneous dendritic cells, and could also result in down-regulation of their contact hypersensitivity (Sugita et al., 2010). Increased NO has been shown to inhibit leukocyte trafficking by the choroid plexus into the central nervous system by down regulation of the epithelial NFκB/p65 signaling pathway. Administration of an NO scavenger alleviated the NFκB/p65 suppression, and restored normal immune cell trafficking into the central nervous system (Baruch et al., 2015). Numerous studies described that the presence of NO suppressed cellular movement and proliferation in various categories of cells, especially, by altering its normal signaling pathways through nitrosylation of various proteins that were essential for directed cellular movement (Maeda et al., 2000; Ciani et al., 2004; Sato et al., 2007; Tsihlis et al., 2011).

There is a paucity of information of the effect of NO on the proliferation and migration of primary cultured stem cells. NO has a highly variable effect on proliferation and migration of stem cells of all categories. In bone marrow-derived endothelial progenitor cells (EPCs), suppression of NO production by the inhibitor, L-NAME, significantly reduced cellular migration, proliferation, and angiogenesis tube formation suggesting that NO is a necessary requirement for these various cellular functions (Lu et al., 2015). Conversely, the directed migration of pluripotent stem cell-derived neural stem cells toward cancer target cells was promoted by the suppression of NO. In this model, suppression of NO improved stem cell tumor tropism (Chen et al., 2013). In multipotent vascular stem cells (MVSCs), proliferation was inhibited by increased NO in a dose dependent manner, and NO further inhibited the differentiation of MVSCs to mesenchymal-like stem cells (Curtis et al., 2014). Exogenous NO was observed to suppress the “collective migration” of primary adult bone marrow stromal cells (BMSCs), but simultaneously enhanced their differentiation potential (Fuseler and Valarmathi, 2012).

In this study, we investigated the effects of short-term and long-term exposure of exogenous NO on single cell migration kinetics, proliferation capacity, and changes in cellular morphology in primary cultured MSCs. Single cell migration kinetics were determined by analyses of the time-displacement curves, in the presence or absence of NO over 24 h. The proliferation capacity was determined by measurement of the mitotic index of the migrating MSCs in the same cultures over the same time period. The changes in cellular morphology were determined by measurement of the fractal dimension of individual MSCs as a function of time, and correlated with cellular displacement in the same cultures and over the same time period.

## Materials and methods

### MSCs isolation, maintenance, and subculture

All animal procedures were carried out in accordance with the guidelines for animal experimentation set forth and approved by Institutional Animal Care and Use Committee (IACUC), College of Veterinary Medicine, University of Illinois at Urbana-Champaign. Primary cultured MSCs were extracted from the bone marrow of young adult 80 g Sprague Dawley®™ SD®™ rats following the previously described protocol (Valarmathi et al., 2008; Fuseler and Valarmathi, 2012). The isolated single cells were expanded further by subcultivation, following three passages the attached bone marrow mesenchymal stem cells were devoid of any non-adherent cell population.

### Immunophenotyping of MSCs by single color flow cytometry

Quantitative analysis for various cell surface markers (Table 1) was performed by single-color flow cytometry using a Coulter® EPICS® XL™ Flow Cytometer as described previously (Valarmathi et al., 2008; Fuseler and Valarmathi, 2012).

**Table 1:** **Primary antibodies used in this study**.

**Primary antibodies**	**Dilutions**	**Source**	**Cell target**
**MSCs CHARACTERIZATION MARKERS**			
CD11b	1:50	BD Pharmingen	Leukocytes
CD31	1:10	Abcam	Endothelial
CD34	1:50	Santa Cruz Biotechnology	Hematopoietic
CD44	!:10	Gene Tex, Inc.	Leukocytes
CD45	1:50	BD Pharmingen	Leukocytes
CD73	1:50	BD Pharmingen	MSCs
CD90	1:50	BD Pharmingen	MSCs
CD106	1:50	BD Pharmingen	Endothelial

### Enrichment of MSCs by magnetic-activated cell sorting (MACS)

Additional purification and enrichment of the cultured MSCs were performed by indirect magnetic cell labeling method using an autoMACS™ Pro Separator (Miltenyi Biotech), as per our previously published method (Fuseler and Valarmathi, 2012). The resultant enriched CD45^−^/CD34^−^/CD90^+^ fractions were subcultured, expanded further, and finally, subjected to flow cytometric analysis (Valarmathi et al., 2008).

### MSCs mattek dish culture and SNAP treatment

The passage four purified and enriched MSCs were seeded at a density of 2 × 10^5^ cells per 10 mm glass bottom MatTek culture dishes, and incubated in a humidified atmosphere of 5% CO_2_ at 37°C for 48 h, before use. The control MSCs and experimental treated MSCs were cultured in complete Dulbecco's Modified Eagle's Medium (DMEM) supplemented with 8% horse serum, 5% newborn calf serum, gentamicin (50 μg/ml), and amphotericin B (250 ng/ml), for further 1–7 days. MSCs experimental and control cultures maintained for 7 days were replenished with fresh medium on every other day (i.e., every 48 h).

### Wound-healing model of cellular migration

The scratch assay (or wound-healing assay) has been employed as a sensitive method to characterize cellular proliferation and migration. The spreading and migration capacity of MSCs were assessed in the absence or presence of the NO donor, S-Nitroso-N-Acetyl-D,L-Penicillamine (SNAP), as described previously (Fuseler and Valarmathi, 2012) with slight modifications.

“Under direct observation of an inverted phase contrast microscope, a linear wound region was created with the tip of a sterile, glass pasture pipette. The pipette was placed directly in contact with the surface of the cover glass and moved across the central region of the confluent monolayer. The sharp edge of the pipette removed cells from the cover slip surface, resulting in a rectangular region that was devoid of cells. This process resulted in essentially straight margins of the wound created in the monolayer of cells (Fronza et al., 2009; Menon et al., 2009). The remaining attached monolayer of cells in the culture dish was washed twice with medium, especially to remove any dead cells and cellular debris, and then, these culture dishes were replenished with 2 ml of fresh medium. The control experimental cultures were maintained in normal medium alone. The test experimental cultures were maintained in medium containing the NO donor, SNAP at a concentration of 200 μM [SNAP was made up in a concentrated stock solution (1.0 mM) and diluted to 200 μM immediately before use],” (Fuseler and Valarmathi, 2012).

In one set of experiments (SN-1), immediately following the creation of the wound in the monolayer, a dish of MSCs was treated with SNAP, and the culture dish was immediately placed on the stage of an inverted microscope and the 24 h time-lapse image acquisition was commenced. The control dishes of MSCs with normal medium alone were imaged and recorded under the same experimental conditions and instrument settings.

In a second set of experiments (SN-2), culture dishes of MSCs were maintained in the presence of SNAP (200 μM) for 7 days, and the controls were maintained in normal medium. At the end of 7 days, wounds were formed in the monolayer, and these cultures were continuously exposed to SNAP during the 24 h period of image acquisition to determine the response and migration capacity of these MSCs. Control MSC cultures were treated and imaged in the same manner with normal medium. In these second series of experiments the control medium (not supplemented with SNAP) and experimental medium (supplemented with SNAP) were changed every 48 h during the 7 day culture period.

### Live cell imaging and analysis

#### Image acquisition

Live cell imaging and Image acquisition set up were exactly followed as per our previously published protocols (Fuseler and Valarmathi, 2012).

“Following creation of the wound area in the MSCs monolayer cultures, the MatTek dishes were immediately mounted on the stage of a Zeiss Axiovert 200M inverted microscope in a Zeiss M200 humidified incubator chamber, and maintained at 37°C and 5% CO_2_ during the course of observations and the collection of images. An appropriate area of the margin of the wound was selected for imaging. Images were collected by time-lapse microscopy using phase contrast optics (10X, N.A. 0.25 PH-1 Achroplan objective) and a CCD camera (Hamamatsu ORCA-ER) controlled by Kinetic Imaging Image Acquisition Software (AQM Advance-6) software at a rate of 10 frames per hour for a total period of 24 h. The incident light during exposure of the image was filtered with 546 nm green interference filter, and exposure times were maintained constant at 23.15 ms for all kinetic cell migration experiments,” (Fuseler and Valarmathi, 2012).

#### Measurement of MSCs cellular movements and displacements

The effect of exogenous NO on directed cellular movement was determined by measurement of the displacement of individual MSCs into the wound region. The changes in the displacement of individual MSCs were determined by frame-by-frame analysis with the AQM Advance-6 software, and were expressed as the mean of the final displacement of the MSCs from the wound margin at 24 h. On an average, 50 cells were measured for each experimental group treated with SNAP (SN-1 and SN-2) and their corresponding control groups.

#### Analysis of the movement of MSCs into the wound zone

Plots of the X–Y displacement of migrating MSCs as a function of time were analyzed to determine the displacement functions that best described cells movement under control conditions and in the presence of exogenous NO.

#### Changes in the fractal dimension of the MSCs during migration in the presence or absence of NO

Changes in MSCs cellular morphology can be quantified by fractal dimension analysis, following as per our previously published protocols (Fuseler and Valarmathi, 2012).

“The wound margin of the monolayer of MSCs appeared as an irregular, complex object composed of many cells. These marginal MSCs, when undergoing migration movement, possessed and continuously expressed cytoplasmic extensions with different levels of resolution (thin and thick filopodia of different dimensions; and lamellipodia also of different dimensions), which were functionally and physiologically similar (self-similar) to the whole object (the leading edge of the MSCs cytoplasm). Under the conditions of these properties, the morphology of individual MSCs that were advancing into the open wound region could be considered fractal objects and their topological dimension, the fractal dimension (*D*), expressed by a non-integer number lying between two Euclidian integer topological dimensions (Grizzi et al., 2005). The values of *D* characterizing the time dependent change in the space filling morphology and degree of chaos associated with the margin or borders of the advancing individual MSCs, therefore were fractional numbers lying between 1 and 2. In these experiments, the value of *D* characterized the complexity or chaos associated with the shape or topological morphology of the individual MSCs localized on the leading margin of advancing monolayer, which were migrating into the wound area. The complexity of the MSCs morphology was represented by the turnover and fluctuation of their cytoplasmic processes as the cells migrated into the wound region. The box counting method (Fernandez and Jelinek, 2001; Grizzi et al., 2005) was used to determine the fractal dimension (D) of the MSCs monolayer. Of the numerous methods for applying fractal analysis to biological and non-biological systems, the box-counting method was most widely used, and provided a general model for determining (*D*) for diverse systems (Fuseler et al., 2007, 2010; Moledina et al., 2011; Wedman et al., 2015). The box-counting method consisted of a grid of boxes of size e superimposed over the image or the region of interest (ROI) of the image or structure, here individual MSCs, and the number of boxes containing any part of the ROI or structure recorded as *N*(*e*). A fractal object expressed a straight line when *Log* [*N*(*e*)] was plotted against *Log* (1/*e*). The box fractal dimension (*D*) could be determined from the slope of the regression line, i.e., (*D*) = *Log* [*N*(*e*)]/*Log* (1/*e*). The values of *D* were determined using HarFA mathematical analytical software (Nezadal et al., 2001; Fuseler et al., 2010). The HarFA software for the 10x images assigned mesh sizes of boxes with e values ranging from 2 to 215 pixels and 10 steps within this range were calculated to generate the *Log* [*N*(*e*)] vs. *Log* (1/*e*) lines to determine D. In these 10x images, one pixel was = 1.7786 μm,” (Fuseler and Valarmathi, 2012).

### F-actin staining and confocal microscopic analysis

F-actin was labeled and visualized with Alexa fluor® 488 Phalloidin (1:200 in PBS, Molecular Probes) as described previously (Fuseler and Valarmathi, 2012). Nuclei were stained with DAPI (4, 6-diamidino-2-phenylindole, 100 ng/ml; Sigma-Aldrich). Images of the stained cells were visualized using confocal microscopes (Zeiss LSM 510 Meta CSLM and/or Zeiss Axiovert 200M Inverted Microscope). Negative controls for staining were also included.

### Descriptors of MSCs actin morphology

The morphometric descriptors of area (A), integrated optical density (IOD), and fractal dimension (*D*) were measured for thresholded actin cytoskeleton of the control, SN-1, and SN-2 MSCs.

#### Isolation and measurement of area of MSCs

In digitized images, fluorescently labeled actin cytoskeletons were isolated as individual regions of interest (ROI) using the HSI (hue, saturation, and intensity) color model of the set color threshold subroutine of MetaMorph 6.1 software. The area of the cell was measured within the ROI, as per our previously published protocol (Fuseler et al., 2006, 2007; Fuseler and Valarmathi, 2012) with slight modifications.

#### Integrated optical density (IOD) of MSCs actin cytoskeleton

“The HSI values were adjusted to isolate the actin cytoskeleton of the MSCs as a thresholded region. From the thresholded region, the area (A) and its integrated optic density (IOD) were measured using the integrated morphometry subroutine of MetaMorph 6.1 software. The images of the individual isolated actin cytoskeletons were converted to a gray scale image for further analysis of its fractal dimension using the free HarFa imaging software (http://www.fch.vutbr.cz/lectures/imagesci/),” (Fuseler et al., 2006, 2007; Fuseler and Valarmathi, 2012).

“When using thresholded boundaries in an image, the integrated optical density (IOD) was defined as the weighted sum of the image histogram in which each term in the histogram was multiplied by the gray value it represented (Walter and Berns, 1986), and was expressed by the following equation:

$$IOD\;\left(T1,T2\right)=\underset{GV=T1}{\sum^{T2}}H(GV)xGV$$

Where, T1 and T2 represent the upper and lower thresholds defining the ROI in the histogram, GV, the gray value of each pixel, and H(GV), the gray level histogram. Values of IOD were calculated directly from the integrated morphometry subroutine of MetaMorph 6.1 software. Using the software's optical calipers, the measurements were refined by setting specific boundary conditions for area and IOD for acceptance of the signal from the individual MSC actin cytoskeleton and to minimize or eliminate the contributions of any non-specific and background staining. This concept of IOD representing the mass or total amount of stained material in an ROI of an image was well-established, and has been previously applied to diverse topics including the translocation of nuclear factor-κB in the nucleus (Fuseler et al., 2006; Rogers and Fuseler, 2007), changes in actin cytoskeleton in cardiac fibroblasts and adult stromal stem cells (Fuseler et al., 2007; Fuseler and Valarmathi, 2012), as well as comparison of the architecture of normal and tumor microvasculature (Fuseler et al., 2010). The IOD measured for the actin cytoskeleton was a representation of the mass and a measurement of the total amount of material in the delineated region. As a mass measurement, the values of IOD provided further information on the structure and distribution of pixels constituting the mass of F-actin that was present within the ROI (Fuseler et al., 2006, 2007; Fuseler and Valarmathi, 2012).”

#### Fractal dimension of MSCs actin cytoskeleton

“The actin cytoskeleton is an irregular and complex object composed of filaments (actin thin filaments, thin actin fibers, and thick actin stress fibers, at different levels of resolution), which are functionally and physiologically similar (self-similar) to the whole object. Because of the complexity of form, the actin cytoskeleton cannot be characterized or defined by regular Euclidean geometry or dimensions. The regular Euclidean dimension assigns an integer to each point or set of points in Euclidean space and includes the familiar geometrical descriptors or numbers: 0 to a point, 1 to a straight line, 2 to a plane surface, and 3 to a volume or three dimensional figures. These integer descriptors are exponents of power functions that describe these objects (Brown et al., 2002). Complex macro- or micro-anatomical structures cannot be analyzed by regular Euclidean geometry, but can be described quantitatively by fractal geometry (Mandelbrot, 1982; Smith et al., 1996). The fractal dimension of complex or irregular structures can be described by non-integer numbers, with values falling between two-integer topological dimensions. These non-integer numbers are described as non-Euclidean and define the fractal dimension (*D*) of an object. The concept of fractals currently provides a useful method to quantify the inherent irregularity or complexity of phenomena (Zhang et al., 2006). In general, a fractal is any rough and irregular object consisting of parts that are in some way similar to the whole. Because of the self-similar conditions, the actin cytoskeleton can be considered a fractal object and its fractal dimension (*D*), expressed by a non-integer number lying between two Euclidian integer topological dimensions (Grizzi et al., 2005). In the case of the two-dimensional images analyzed in this study, the values of *D* characterizing the actin cytoskeleton are therefore fractional and lie between the Euclidian integers of 1 and 2. This further implies that the actin cytoskeleton lying in a single optical section will express a value of *D* >1 because it is an object more space-filling than a straight line, and <2 because the object does not completely fill the plane it occupies,” (Fuseler et al., 2006, 2007; Fuseler and Valarmathi, 2012).

“The box-counting method has been the most widely used and general model for applying fractal analysis to biological and non-biological systems and is expressed by the formula:

$$\begin{array}{l} D=\underset{e\to 0}{\lim}\frac{LogN\left(e\right)}{Log\left(1/e\right)} \end{array}$$

The box-counting method consists of a grid of boxes of side length size (*e*) superimposed over the image of the structure and *N*(*e*) the smallest number of boxes of side (*e*) required to cover the surface or outline of the object completely (Grizzi and Dioguardi, 1999; Dioguardi et al., 2006; Fuseler et al., 2007). Since a zero limit cannot be applied to a biological object, here the actin cytoskeleton, the fractal dimension is determined as the slope of a regression line when *Log* [*N*(*e*)] is plotted against *Log* (1/*e*) i.e., *D* = *Log* [*N*(*e*)]/*Log* (1/*e*),” (Fuseler et al., 2006, 2007; Fuseler and Valarmathi, 2012).

“From the images of previously isolated and thresholded actin cytoskeleton, the *D*-values of the ROI were determined using HarFA (full version 5.5.31) software (Nezadal et al., 2001). The thresholded images of actin cytoskeleton were assigned mesh sizes of boxes with (*e*) values ranging from 2 to 215 pixels and 30 steps within this range were calculated to generate the *Log* [*N*(*e*)] vs. *Log* (1/*e*) plots, the slopes of which determine the *D*-values. A minimum of 100 cells were analyzed from each experimental group,” (Fuseler et al., 2006, 2007; Fuseler and Valarmathi, 2012).

### Mitotic counts of migrating MSCs

The time lapse images (for the entire 24 h collection period) were examined frame-by-frame, and cells that were clearly under mitosis were identified. The dividing cell was isolated from the total image. From all the frames containing the dividing cell from interphase to separation of the daughter cells were collected. The individual cell was outlined along its margin as a region of interest (ROI) and isolated from the back ground. The isolated cell was thresholded. The morphometric descriptors were then measured for the dividing cell in each of those isolated frame images. The most sensitive of the morphometric parameters measured the cell area and integrated optical density (IOD) of the gray scale of the pixels comprising the image of the cell within the ROI, and were the most sensitive to change. The ratio of IOD/A yielded a curve that was typical for cells undergoing mitosis. Cells that were rounded up but did not produce this type of descriptive curve were excluded from the count of dividing cells in the recording. Cells that were exposed to NO donor did not undergo division. Cells that were exposed to NO and were rounded up did not generate this typical IOD/A curve describing mitosis.

### Statistical analysis

For all the experimental data, the differences in the mean between the test groups and their corresponding control groups were determined by applying one-way ANOVA and Student's *t*-test (Sigma-Stat v11). In all cases, *p* < 0.05 were considered statistically significant.

## Results

### Phenotypic characterization of undifferentiated postnatal MSCs

**Figure 1:**
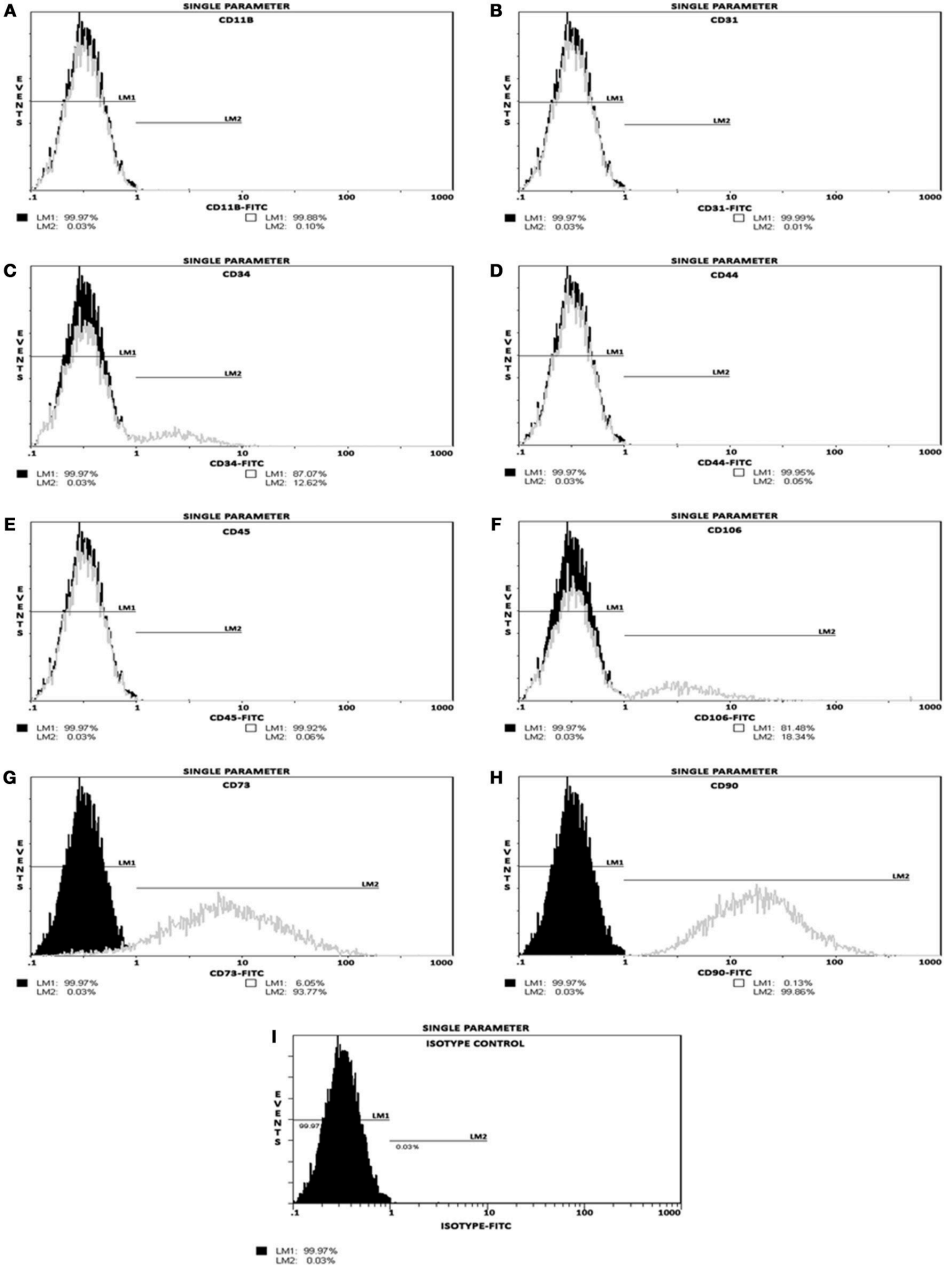
**Postnatal MSCs surface marker expression**. Analysis of positive and negative cell-surface antigen expression in a pool of expanded and passage 3 postnatal bone marrow-derived MSCs, by single color flow cytometry. **(A–F)** Histograms validating percentage expression of MSC-associated negative markers. The intensity and distribution of cells stained for endothelial and hematopoietic cell-surface antigens, such as CD11b, CD31, CD34, CD44, CD45, and CD106 (gray, open peaks) were not significantly different from those of isotype control (**I**; black, shaded peaks). **(G,H)** Histograms validating percentage expression of MSC-associated positive markers. The fluorescent intensity was greater (moved to right) when MSCs were stained with CD73 or CD90 surface antigens. These results indicated that these cultures contained a near-pure population of MSCs as defined by the minimal criteria for MSC surface antigen immunophenotyping.

Phenotyping of postnatal MSCs for various cell surface antigens by single color flow cytometry validated that the fluorescent intensity and distribution of the cells stained for endothelial and hematopoietic cell-surface antigens, such as CD11b, CD31, CD34, CD44, CD45, and CD106 were not significantly different from those of isotype controls (Figures 1A–F,I). In contrast, MSCs exhibited a high expression of CD90 (99.86%) and CD73 (93.77%) surface antigens (Figures 1G,H). These results indicated that the cultures were devoid of any bone marrow-derived hematopoietic stem and/or progenitor cells as well as matured endothelial cells (Table 1), and contained only a near-pure population of MSCs as defined by the minimal criteria for MSC surface antigen immunophenotyping (Dominici et al., 2006; Valarmathi et al., 2008).

### MSCs migration into the wound area

**Figure 2:**
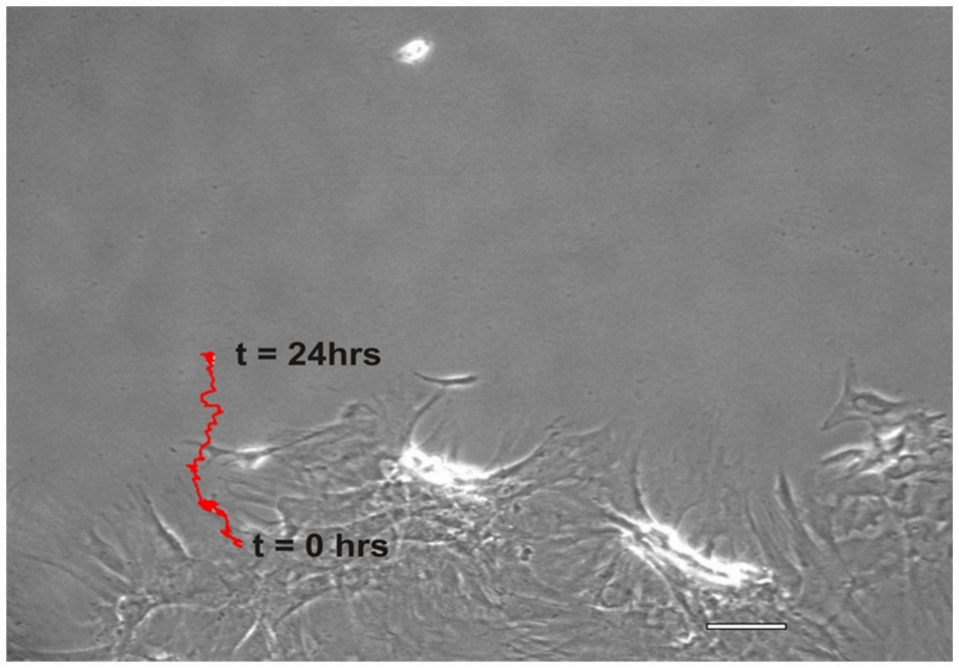
**Representative migration path of a MSC from the wound margin into the open wound area over a period of 24 h, *t* = 0 to *t* = 24 h**. The red trace distance was the total length and shape of the migration path, i.e., the course typically taken by an individual MSC. (Scale bar = 100 μm).

**Figure 3:**
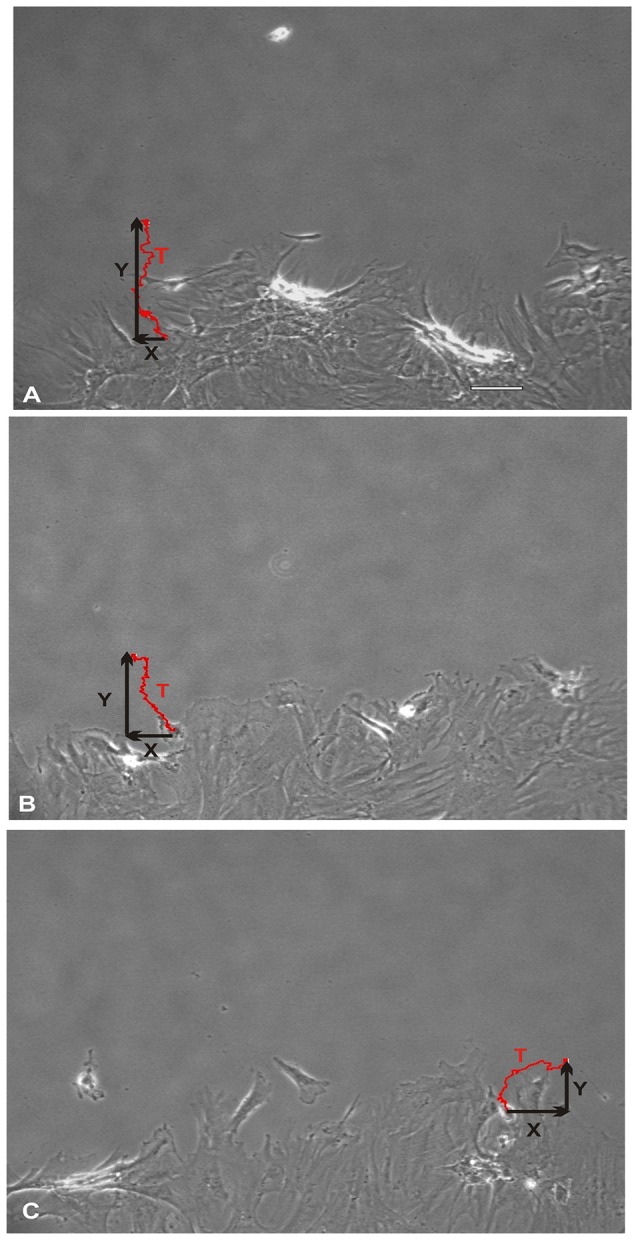
**Total direct movement (in Y direction) and lateral movement (in X direction) of MSCs into the wound area**. Red line trace showing the typical migration path of a MSC over a period of 24 h. **(A)** Control MSCs exhibited their maximum cellular movement directly into the wound zone. **(B)** Whereas, MSCs that were treated with nitric oxide (NO) immediately upon wound formation (group SN-1) suppressed their maximal migration distance (Y). In addition, these MSCs exhibited an increase in their lateral movements (X). **(C)** The MSCs that were pretreated with NO for 7 days prior to wound formation (group SN-2), not only had maximal effect on suppressing their migration into the wound zone (Y) but also further increased their lateral movements (X). (A, scale bar = 100 μm).

**Figure 4:**
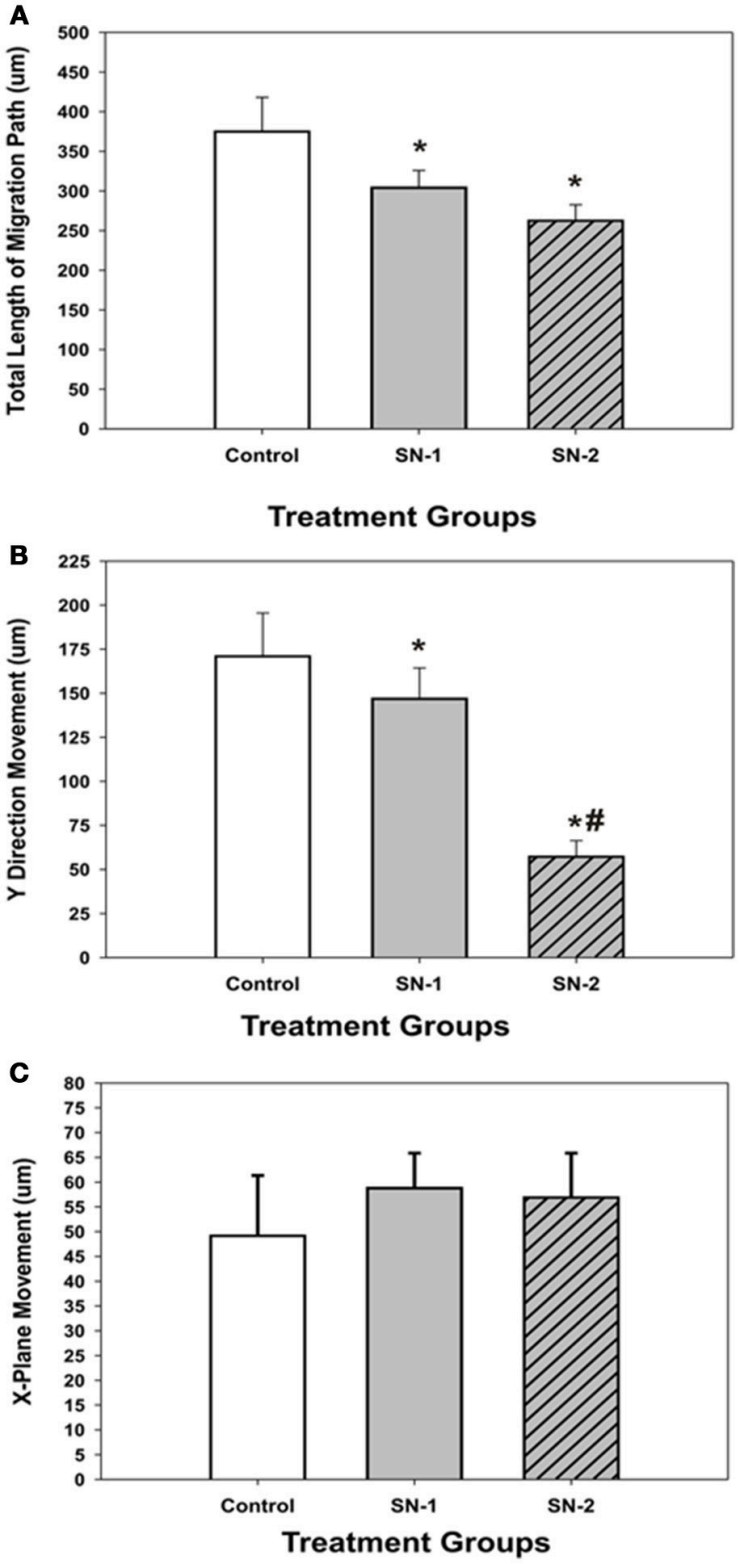
**Comparison of the effect of NO on direct and lateral movements of MSCs into the wound area. (A)** Comparison of the total length (T) of the MSC migration path. ^*^difference from controls. The control T was greater than both SN-1 T (*P* = 0.036) and SN-2 T (*P* = 0.012). SN-1 T was not different from SN-2 T (*P* = 0.141). **(B)** Comparison of the magnitude of MSC movements in the linear, Y plane. Direct movements into the wound zone. ^*^difference from control. The control Y was greater than SN-1 Y (*P* = 0.033) and SN-2 Y (*P* < 0.001). #difference from SN-1. SN-1 Y was greater than SN-2 Y (*P* < 0.001). **(C)** Comparison of the magnitude of MSC movements in the lateral, X plane. All movements, left or right were considered as positive. There was no difference in the magnitude of MSC movements in the X plane for the control X, SN-1 X, and SN-2 X. (*P* < 0.05).

**Figure 5:**
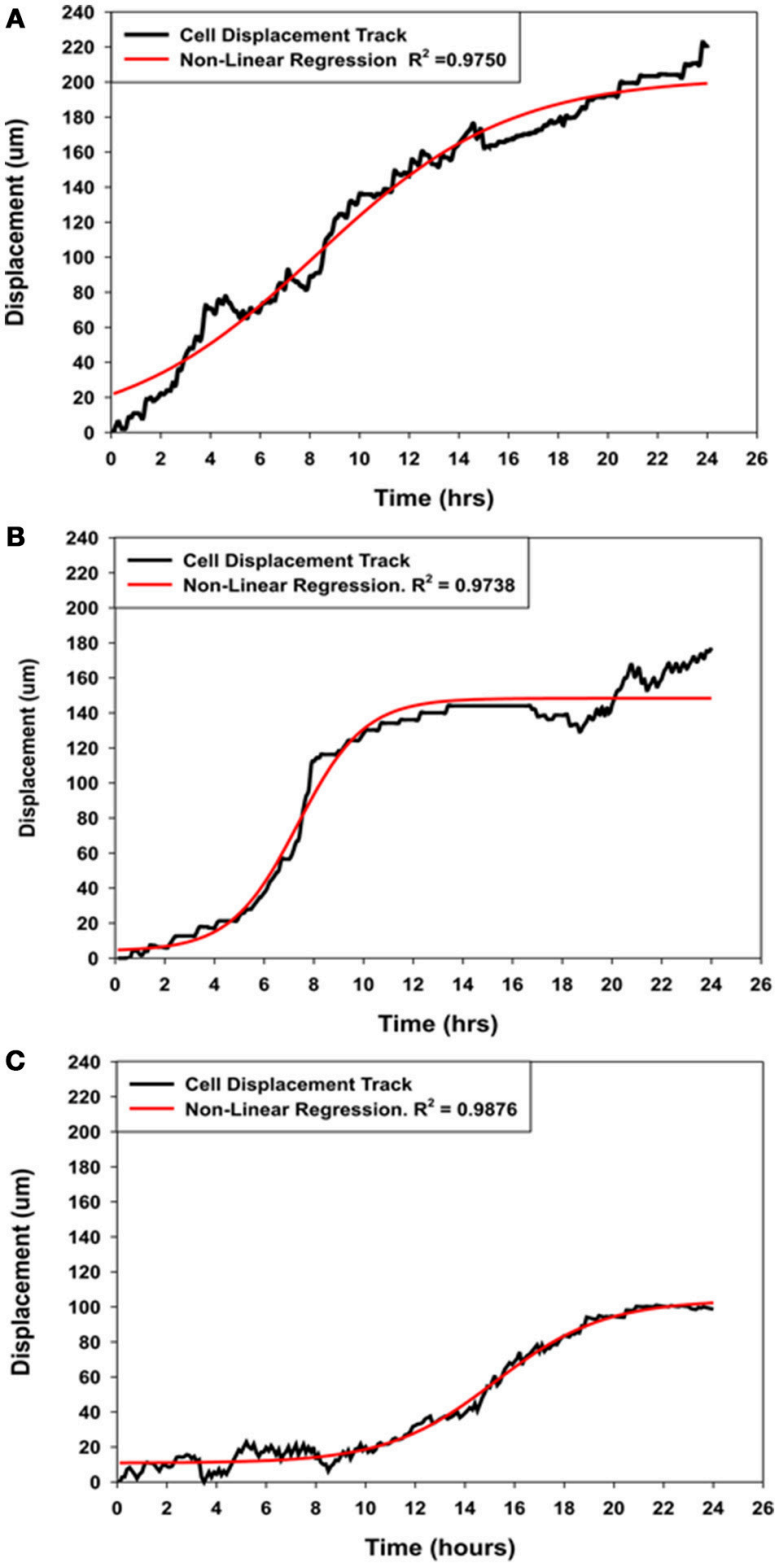
**Comparison of the displacement kinetics of MSCs over 24 h of migration into the wound zone. (A)** Typical displacement kinetic curve for control MSCs. **(B)** MSCs that were migrating in the immediate presence of NO (Group-SN-1) exhibited initial movement followed by suppression of their directed movement into the wound zone. **(C)** MSCs that were pretreated with NO donor for 7 days (Group-SN-2) prior to wound formation exhibited suppression of their initial movement followed by a brief period of directed migration, and finally cessation of their cellular movement. The displacement curves for all three groups could be described by non-linear regression analyses (solid red line curves).

NO suppressed MSCs migration into the wound zone. The extent of migration suppression appeared directly proportional to the time of exposure to NO. In the wound model of cellular migration, cells at the margin of the wound preferentially migrated into the cell-free zone without the addition of attractants. The typical migration track of control MSCs (Figures 2, 3A) (Video 1, Supplementary Material) exhibited primarily directed movement into the open wound zone. Exogenous NO applied immediately following wound formation (group SN-1) suppressed movement into the wound zone (Figures 3B, 4A) (Video 2, Supplementary Material), and significantly shortened the length of the migration path in the Y plane (Figure 4B). However, there was an increase in lateral movement in the X plane, but this was not significantly different from that seen in the control groups (Figure 4C). In the presence of NO, there was a lag time of up to 2 h before the MSCs begin their directed movement into the wound zone. The MSCs then exhibited movement at essentially constant velocity between 4 and 8 h, after which directed movement rapidly declined, and the directed movement ceased in many cells (Figure 5B). In cells, in which movement had ceased, there was continued active membrane movement and ruffling. The MSCs which had stopped movement neither exhibited rounded-up morphology nor detached from the substrate, or expressed any indications of undergoing cellular degeneration.

Pretreatment of the MSCs with SNAP for 7 days prior to wound formation (group SN-2) had the most dramatic effect on directed MSCs movement (Figure 3C) (Video 3, Supplementary Material). The total migration path length was significantly shortened compared to the controls and SN-1 groups (Figure 4A). Numerous cells had minimal directed movement in the Y direction, and remained in a stationary state while expressing extensive membrane ruffling combined with extension and contraction of multiple cytoplasmic processes. The most significant effect of NO on this group was marked suppression of movement in the Y plane into the wound zone (Figure 4B). Interestingly, the MSCs of this group exhibited the same measure of lateral movement in the X plane as seen in the control and SN-1 groups (Figure 4C).

### Displacement coefficient analysis

The displacement curve describing the movement of the control MSCs into the wound zone was essentially linear with minor oscillations, but can also described by a four parameter sigmoid non-linear expression [*y* = *y*_*o*_ + *a*/(1+exp(−(*x*−*x*_*o*_)/*b*))], where *x* was time in hours and *y* was the displacement distance in μm. This expression was applied because it also elegantly described the displacement kinetics of the control MSCs, and both experimental groups of MSCs treated with NO for short (SN-1) and long (SN-2) periods of time, whereas linear regression did not. The typical migration displacement of control MSCs, illustrated in Figure 5A. The cells began directed movement immediately, and maintained essentially constant velocity over the 24 h of the experiment.

The immediate application of NO to migrating MSCs (group SN-1) had an instantaneous effect on cellular migration. The initial movement of MSCs between 1 and 5 h was slow. The migration rate of the MSCs then increased for between 5 and 6 h after which the cell began to slow and ceased their directed movement (Figure 5B). The stationary MSCs continued to exhibit active membrane ruffling, and the extension and contraction of filopodia. Some of these cells also exhibited limited random movements including reverse directions. These suggested that NO has an accumulative effect on the MSCs to follow the directed signal pathway created by the wound area.

The pre-treatment of MSCs with NO (group SN-2) had a more severe effect on cellular migration, and was clearly illuminated by the displacement kinetics. Following formation of the wound, the MSCs exhibited minimal or no directed movement into the wound area between 8 and 10 h. Following this time period there were limited directed movement of MSCs into the wound zone (Figure 5C). The MSCs maintained slow directed movement for about 10 h when directed cellular movement began to cease. Upon stopping, these MSCs did not depict any round-up in morphology, indicating lack of cellular degeneration, but continued to exhibit very active membrane movement and ruffling. This suggested that long term exposure to NO altered the capacity of the MSCs to undergo directed movement, but not the cytoskeletal elements associated with cellular and membrane movement.

**Figure 6:**
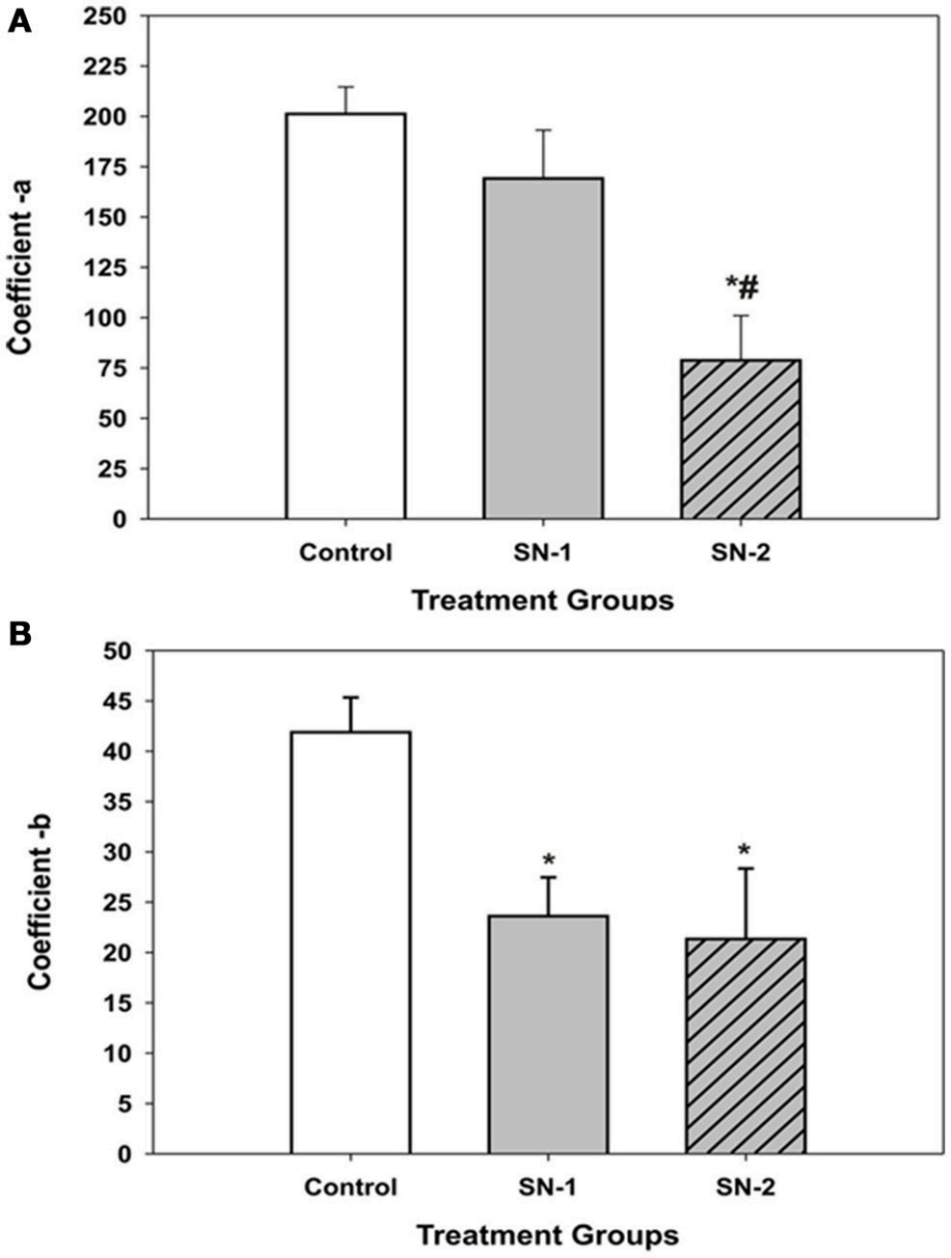
**Comparison of the displacement coefficients of the non-linear expression [*y* = *y*_*o*_ + *a*/(1+exp(–(*x*–*x*_*o*_)/*b*))], which described the movement of MSCs into the wound area in the presence or absence of NO. (A)** The coefficient ***a***, which appeared to be associated directly with cell movement. This value decreased proportionally with length of exposure time to NO. The value of ***a***, for the SN-1 group was decreased but not significantly from that of the controls (*P* = 0.52). However, for the SN-2 group the value of ***a***, was significantly less than that of both the control and SN-1 group (^*^different from controls, *P* < 0.001; #different from SN-1, *P* < 0.001). **(B)** The coefficient ***b***, which appeared to be associated with time kinetics of movement of the MSCs significantly decreased in the presence of NO. Both the NO treated groups were significantly less than the control group [^*^SN-1 and SN-2 < controls (*P* < 0.001)]. The ***b***-values for SN-1 and SN-2 were not different from each other (*P* = 0.101).

The coefficients of the four parameter sigmoid equation which described the displacement of the MSCs into the wound zone as a function of time provided insight into the possible mechanism of how NO suppressed cellular movement. The coefficient, **a** appeared to be associated directly with cell movement and decreased proportionally with length of exposure to NO (Figure 6A). The longer the MSCs were exposed to NO, the greater was suppressive effect on directed migratory movement. Interestingly, the coefficient, **b** which appeared to be associated with time kinetics of movement significantly decreased in the presence of NO to the same value for both the SN-1 and SN-2 groups. The coefficient **b** was the same value for both short and long term exposure to NO (Figure 6B). This would suggest that the cellular mechanism associated with time of movement was altered very early following initial exposure to NO, and the effect of NO on this mechanism was not cumulative. This alteration can occur equivalently in actively moving and stationary MSCs. Once altered the value of **b** remained constant and was not further deceased by continuous exposure to NO, suggesting saturation of this rate of movement controlling site. This component of the suppression of MSCs movement may be the result of the suppression of activity of specific sites yet to be determined by nitrosylation, for example.

### MSCs mean migration velocity

**Figure 7:**
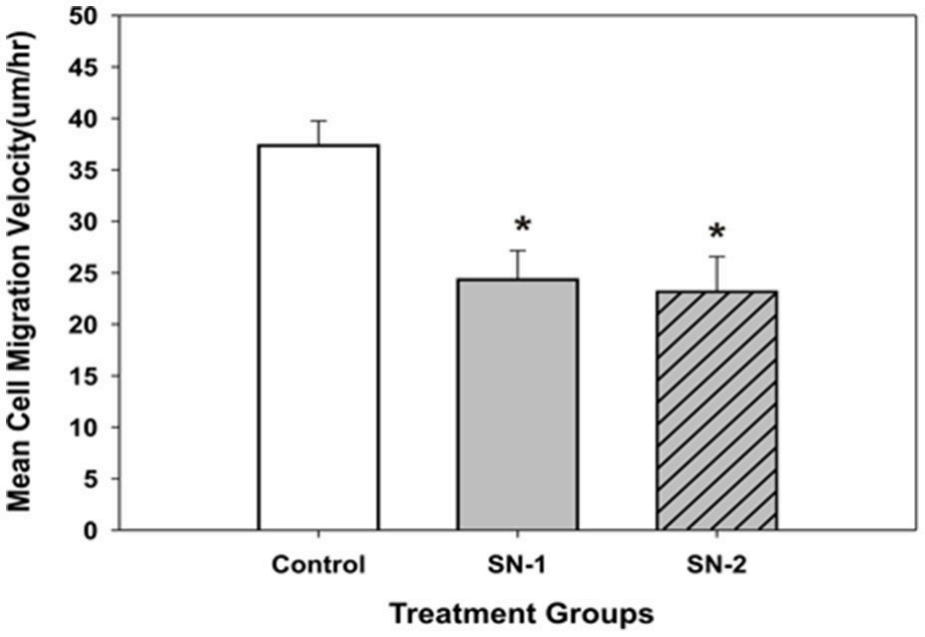
**The mean migration velocity of MSCs got reduced in the presence of NO**. The effect of NO on mean migration velocity of MSCs appeared to be non-cumulative. Both short- and long-term exposure of NO on these cells had the same effect. The mean velocity of the NO treated groups was significantly less than that of the control group [^*^SN-1 < controls (*P* = 0.001) and ^*^SN-2 < controls (*P* = 0.002)]. The observed mean velocity of MSCs in SN-1 and SN-2 groups was not different from each other (*P* = 0.887).

The mean velocity of migrating MSCs was suppressed by exogenous NO. In both groups of NO treated MSCs, the presence of NO significantly reduced the mean migration velocity of the same order of magnitude (Figure 7). This would indicate that the control pathways responsible for the velocity of cellular movement can be immediately affected by NO and the effect of NO on cellular velocity was not cumulative.

### Fractal dimension (*D*) analysis of MSCs migration

**Figure 8:**
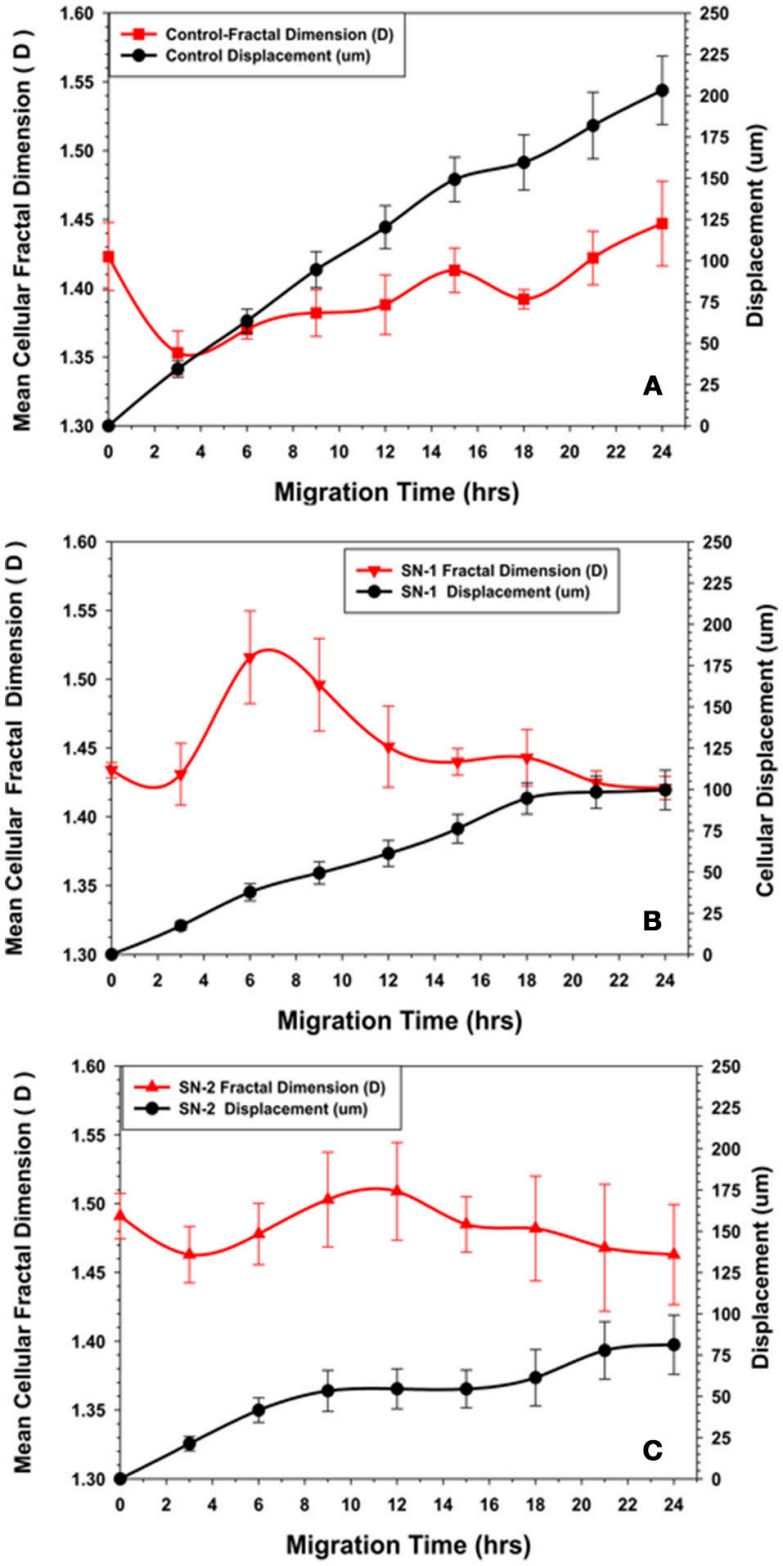
**Comparison of changes in fractal dimension of MSCs with their displacement kinetics in the presence or absence of NO. (A)** Control MSCs. The fractal dimension (*D*) of the migrating MSCs was initially decreased during the first 2–3 h of migration, suggesting reorganization of the cytoskeleton to a more ordered state during this time. The subsequent increase in (*D*) to an essentially steady state value with minor oscillations suggested a certain degree of disorder of the cytoskeleton maintained during directed migration into the wound area. **(B)** Group SN-1. MSCs that were treated with NO at the onset of migration, the fractal dimension (*D*) started at the same value as seen for the controls, then immediately decreased during the first 2–3 h of migration, suggesting reorganization of the cytoskeleton to a more ordered state during this time period. Subsequent slow migration was accompanied by a significant increase in (*D*). This suggested that NO affected migration by increasing disorder or chaos associated with the MSC's cytoskeleton. The (*D*) value then declined to a steady state value similar to that seen at time = 0, when the MSCs ceased their movement all together. **(C)** Group-SN-2. MSCs that were pretreated with NO for 7 days prior to migration revealed a significantly elevated (*D*) value before the start of migration i.e., at time = 0. There was a minor decrease in (*D*) as these cells began their cellular movement as seen in the other groups. During the phase of limited migration of this group, the (*D*) value increased slightly then declined to an elevated steady state value as the MSCs ceased their directed movement.

Cellular migration was accomplished by the cyclic extension of cellular membrane processes in a preferred direction. The membrane processes vary in size, shape, and magnitude; and could be considered fractal structures and the cell as a whole a fractal object (Fuseler and Valarmathi, 2012). The changes in the fractal dimension (*D*) of the MSCs during migration was uniquely depending on time of exposure to the NO donor. In the first 2 h of migration, the (*D*) values for control MSCs, initially decreased, indicating that the morphology of the cells became more regular with the production of fewer random processes. All marginal membrane cytoplasmic projections were less chaotic and presumably associated with movement directed in the Y plane of the wound zone. As the MSCs progressively migrated, the cellular (*D*) began to increase after 4 h. The (*D*) values slowly increased to an essentially a steady state value by 24 h with minor oscillations (Figure 8A). This pattern of changes in (*D*) associated with the relatively constant migration velocity was considered to be characteristic of normal migration of MSCs in this model.

The immediate application of NO donor (group SN-1) at the time of wound formation had dramatic effect on MSC's migration. Over the first 2 h of migration, the (*D*) value increased in a manner similar to that seen in the controls, but to a lesser extent. Between 4 and 8 h the (*D*) value significantly increased, then slowly declined reaching steady state conditions between 16 and 24 h (Figure 8B). This suggested that the effect of NO may occur during this time altering both cellular morphology and the mechanism controlling the rate of cellular movement. When MSC migration slowed to a stop between 18 and 24 h there was no major changes in (*D*). The mean velocity of the migrating MSCs between 0 and 18 h was significantly less than that of the controls over the same time period.

Pretreatment of the MSC monolayer for 7 days prior to wound formation (group SN-2) had the greatest inhibitory effect on MSC migration and altered morphology. The initial (*D*) value for the MSCs was higher than in the controls and group SN-1 MSCs. The (*D*) values remained high and fairly constant over the 24 h of MSC migration into the wound zone. The directed migration of the MSCs ceased between 8 and 10 h following creation of the wound zone. There was some evidence of continued directed movement between 18 and 24 h, but this was minimal and not significant (Figure 8C). The mean velocity of the MSCs between 0 and 8 h was the same as that seen for MSCs treated with NO for 24 h. This suggested that the presence of NO, either in long or short term exposure was sufficient to reduce cellular velocity to the same values. The continued presence of NO apparently did not have a cumulative effect on cell velocity. MSCs moved slower, then stopped moving. The time in which MSCs stopped moving appeared to be directly dependent on time of exposure to NO.

### NO treatment on MSCs F-actin cytoskeleton morphology and fractal dimension (*D*)

**Figure 9:**
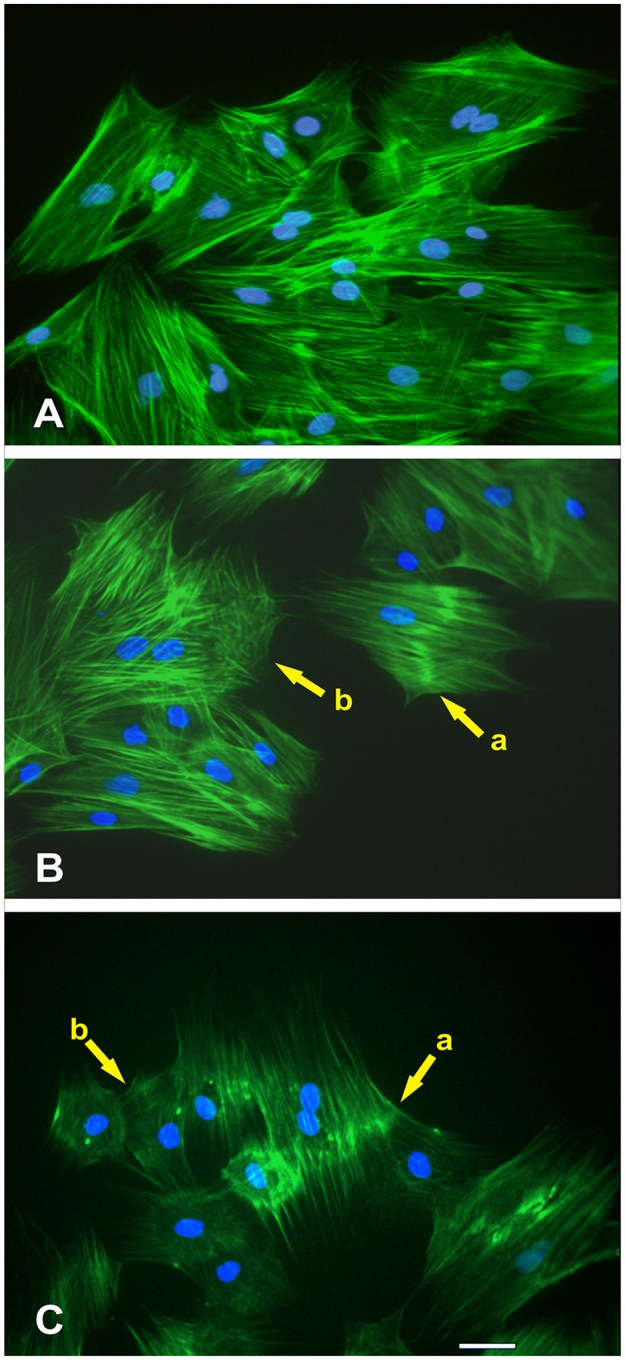
**Effect of NO treatment on MSCs F-actin cytoskeleton morphology. (A)** Typical F-actin cytoskeletal morphology of control MSCs (untreated with NO) that were migrating at the wound margin 24 h after formation of the wound in the monolayer. **(B)** At the wound margin, the actin cytoskeletal morphology of MSCs that were treated immediately with NO exhibited distinctive changes. These structural changes were manifested either as a thickening of the actin fibers in the central region of the cell (yellow arrow, cell-a) or disruption of the cytoskeleton (yellow arrow, cell-b). The nuclei of these cells were intact. **(C)** The long-term treatment of MSCs with NO revealed not only the continued manifestation of the thickening of the actin fibers in the central region of the cell (yellow arrow, cell-a) but also displayed more extensive disruption of the actin cytoskeleton (yellow arrow, cell-b). Besides, the cells became smaller in appearance but retained their stellate configuration. The nuclei of these cells were also intact.

**Figure 10:**
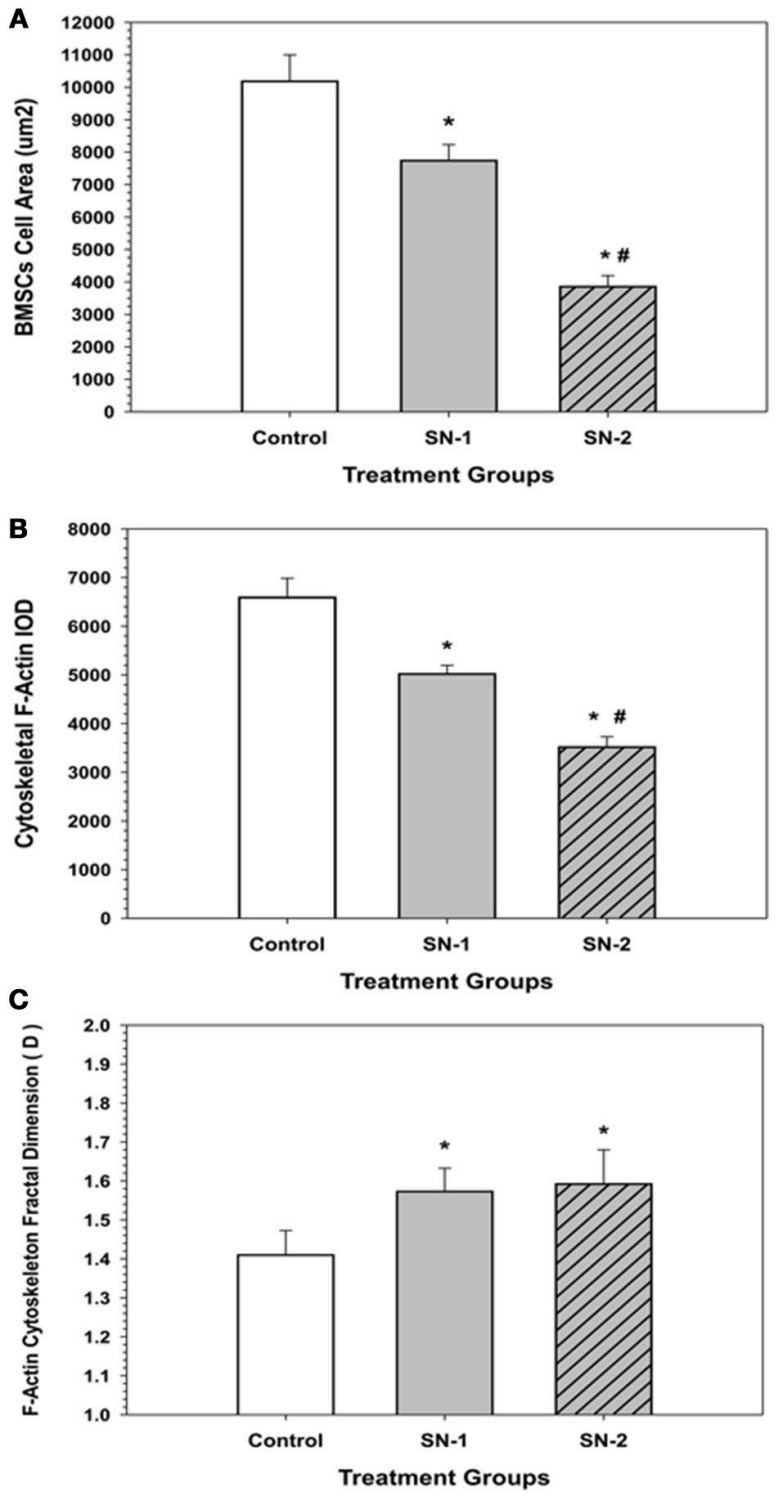
**F-Actin fractal dimension (*D*) got elevated with NO treatment**. In the presence of NO, MSCs demonstrated an elevated level of chaos and disorganization of their actin cytoskeleton. (^*^significantly different from control, *P* < 0.05. There was no difference in D between SN-1 and SN-2). Response of MSCs' actin cytoskeleton to NO treatment. **(A)** Exposure of NO resulted in diminution in cell size. However, MSCs were not rounded up but maintained their stellate configuration. The measured cellular area of MSCs, both in the SN-1 and SN-2 groups were significantly smaller than those of the controls (^*^P < 0.05). The SN-2 MSCs were smaller than the SN-1 cells (#*P* < 0.05). [CT > SN-1, *P* = 0.011; CT > SN-2, *P* < 0.001; SN-1 > SN-2, *P* < 0.001^*^]. **(B)** When the mass of the actin cytoskeleton was measured by the IOD, the F-actin fluorescence was progressively decreased with continued exposure to NO, i.e., the presence of NO reduced the amount of F-actin that was present in the MSCs. The measured F-actin IOD of MSCs, both in the SN-1 and SN-2 was significantly smaller than those of the controls (^*^P < 0.05). The F-actin of SN-2 MSCs were significantly less than the SN-1 cells (#*P* < 0.05). [CT > SN-1, *P* = 0.033; CT > SN-2, *P* < 0.001; SN-1 > SN-2, *P* < 0.001]. **(C)** MSCs, which were exposed to NO, the fractal dimension (*D*) of the F-actin was significantly increased both in the SN-1 and SN-2 treatment groups. The presence of NO induced disorder in F-actin cytoskeleton, and resulted in suppression of directed movement of the cells. The fractal dimension (*D*) values of MSCs in the SN-1 and SN-2 were significantly greater than those of the controls (^*^P < 0.05). The (*D*) values for MSCs in SN-1 and SN-2 were not significantly different (*P* > 0.05). [SN-1 > CT, ^*^P < 0.001; SN-2 > CT, *P* < 0.001; SN-1 = SN-2, *P* < 0.863].

The morphometric descriptors of area (A), integrated optical density (IOD), and fractal dimension (*D*) were measured for thresholded actin cytoskeleton of the control, SN-1, and SN-2 MSCs (Figures 9, 10).

**Figure 11:**
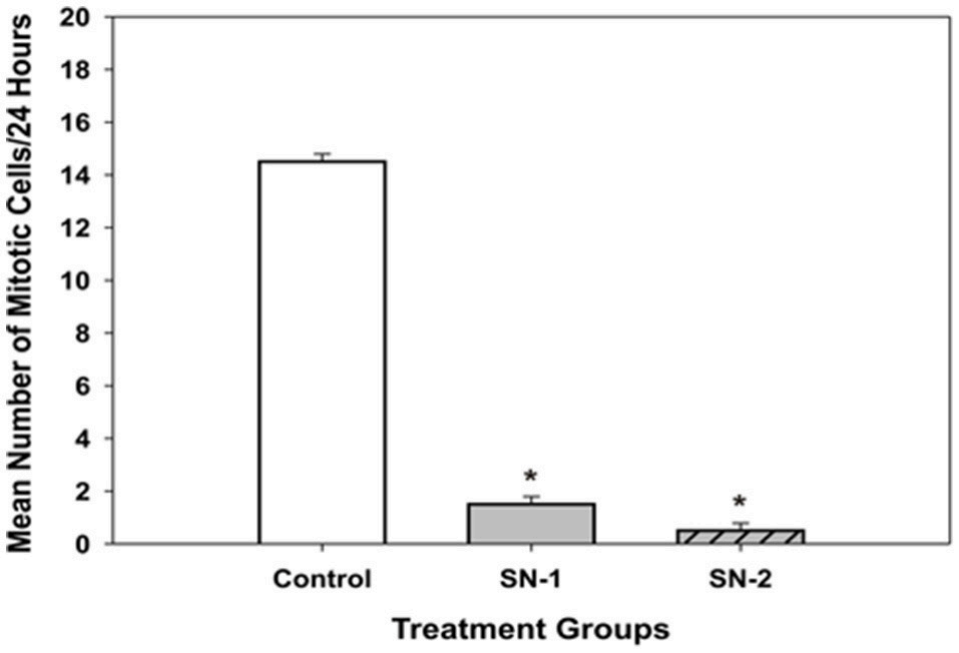
**Mitotic cell division in MSCs got inhibited by NO**. On examination, the presence of actively dividing cells (i.e., cells that were under mitosis) in the NO treated groups were significantly less than that seen in the control group [^*^SN-1 < controls (*P* = 0.029) and ^*^SN-2 < controls (*P* = 0.021)]. Mitosis of MSCs in SN-1 and SN-2 groups was suppressed equally, and was not different from each other (*P* = 0.114).

### Morphological descriptors of MSCs F-actin cytoskeleton

At the wound margin, compared with the controls (Figure 9A), the actin cytoskeletal morphology of MSCs that were treated immediately with NO exhibited distinctive changes. These structural changes were manifested either as a thickening of the actin fibers in the central region of the cell (yellow arrow, cell-a) or disruption of the cytoskeleton (yellow arrow, cell-b; Figure 9B). The nuclei of these cells were intact. The long-term treatment of MSCs with NO revealed not only the continued manifestation of the thickening of the actin fibers in the central region of the cell (yellow arrow, cell-a) but also displayed more extensive disruption of the actin cytoskeleton (yellow arrow, cell-b). Besides, the cells became smaller in appearance but retained their stellate configuration (Figure 9C). The nuclei of these cells were also intact.

### Fractal dimension (*D*) analysis of MSCs F-actin cytoskeleton

In the presence of NO, MSCs demonstrated an elevated level of chaos and disorganization of their actin cytoskeleton. Exposure of NO resulted in diminution in cell size. However, MSCs were not rounded up but maintained their stellate configuration (Figure 10A). When the mass of the actin cytoskeleton was measured by the IOD, the F-actin fluorescence was progressively decreased with continued exposure to NO, i.e., the presence of NO reduced the amount of F-actin that was present in the MSCs (Figure 10B). MSCs, which were exposed NO, the fractal dimension (*D*) of the F-actin was significantly increased both in the SN-1 and SN-2 treatment groups. The presence of NO induced disorder in F-actin cytoskeleton, and was resulted in suppression of directed movement of the cells (Figure 10C).

### NO suppresses mitosis in migrating MSCs

**Figure 12:**
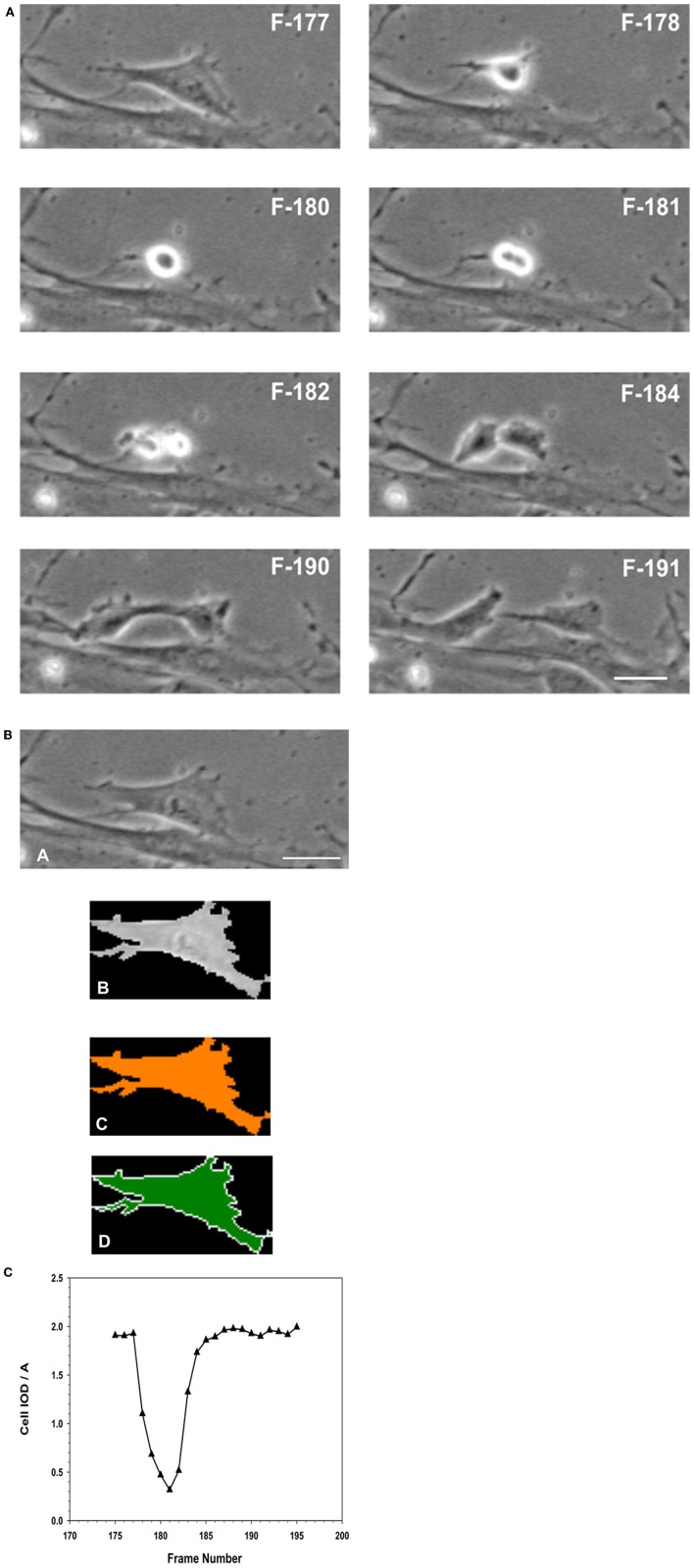
**(A)** Analysis of Mitosis Pattern in Migrating MSCs. Typical pattern of mitosis observed in control MSCs that were migrating in the wound healing model of cellular migration. The F numbers inscribed on the images were the frame numbers from the corresponding time-lapse experimental recording. Frames (images) were captured at a rate of 10 frames per hour (1 frame/6 min) by phase contrast live cell imaging for 24 h. Dividing cells were isolated and morphometric descriptors characterizing a cell transiting through mitosis were determined using Meta Morph image analysis software. When the flattened interphase cell (F177) transitioned into mitosis, the cell progressively rounds up, became smaller and more retractile (F178–F180). The cell then divided into two daughter cells (F181–F182), which progressively flattened (F184–F190) out to resemble the morphology of the original or mother cell (F191). **(B)** Isolation of a single cell from the image, to determine its morphological descriptors characterizing cell division. **(A)** Phase contrast image of the cell was isolated from the image frame. **(B)** Isolation of the cell as a region of interest (ROI) from the surrounding background. **(C)** Thresholding of the isolated cell for measurements. **(D)** Measurements were performed within the ROI, for determination of morphometric parameters characterizing the cell as it progresses through cell division. (Scale Bar = 20 μm). **(C)** Changes in morphology associated with cell division were best described as the changes in the integrated optical density (IOD), and the area (A) of the isolated ROI of the cell, expressed as IOD/A. When the cell entered mitosis, the IOD/A decreased through reaching a minimum as the cell began to elongate forming the two daughter cells. The IOD/A then continued to increase as the daughter cells matured and spread out to resemble the original cell. At the end of this period the IOD/A values were the same as those for the parent cell.

**Figure 13:**
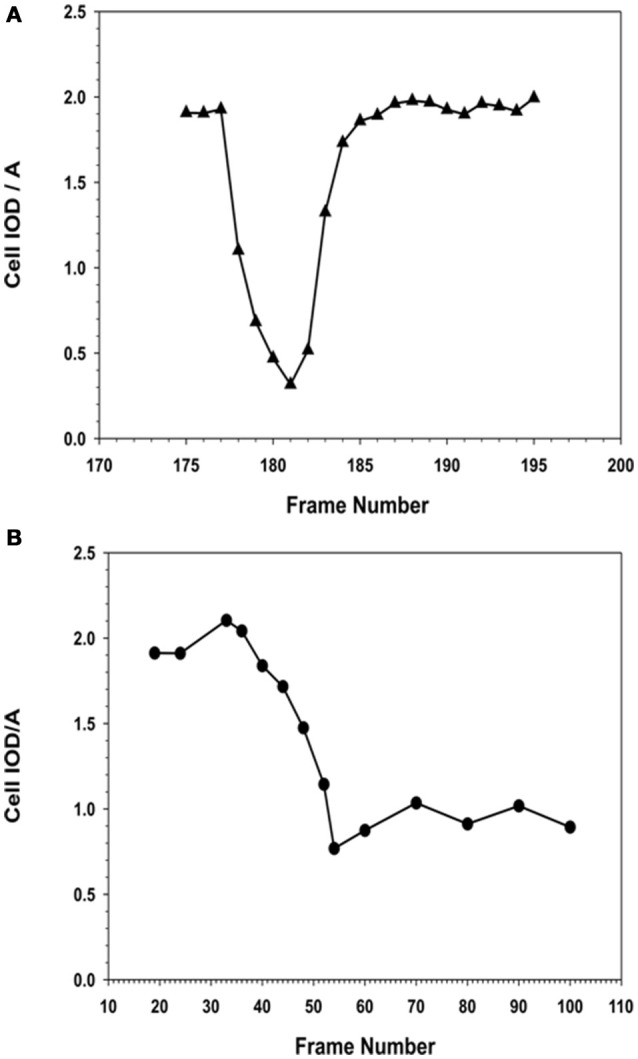
**Comparison of morphometric descriptor, IOD/A for a MSC cell division in the absence or presence of NO. (A)** Cell division in the absence of NO. MSCs that were not exposed to NO, i.e., the normal cell was able to complete its cell division rapidly, between 30 and 45 min, to form two daughter cells. The observed IOD/A descriptor values were same in the case of daughter and the original parent cell. The time from the onset of cell division to maximal rounding of the cell before telophase was between 30 and 36 min. **(B)** Cell division in the presence of NO. MSCs that were exposed to NO, the cell began to round up when it entered mitosis. The process of rounding up was slower than the corresponding control cells, and took up to 120 min to reach the minimal IOD/A values. In the presence of NO, the cells were able to remain rounded up as defined by the continuous expression of the low IOD/A values for the cell. The cell remained rounded up and no daughter cells were formed.

In the presence of SNAP, mitosis of MSCs was essentially inhibited. During the 24 h period of observation, in both the SN-1 and SN-2 groups only 1 or 2 cells were observed to undergo cell division. Over the same time period, mitosis in the control MSCs were regular and significantly (*P* < 0.05) greater (Figure 11). This would indicate that exogenous NO can directly or indirectly affect the organization and function of the mitotic spindle or suppress the cell's ability to enter mitosis. In addition, the control cell divided into two daughter cells, which progressively flattened out to resemble the morphology of the original or mother cell (Figures 12A–C). Moreover, the data suggested that NO retarded the cells entry into mitosis and further prevented the formation of the two daughter cells. In the presence of NO, the cells persisted in a rounded up morphology and did not spread out as daughter cells or as a single bi- or multi- nucleated cell (Figure 13). The rounded up cells were occasionally detached from the substrate.

## Discussion

Here, we report an analysis of the migration of postnatal stem cells, MSCs, undergoing directed cellular movement in a reproducible wound-healing model of cellular migration. This is a further quantitative description of this important trait of adult stem cells at single cell level. We also have demonstrated that NO has a suppressive effect on MSCs proliferation and cell division. As previously demonstrated, MSCs have the intrinsic potential to respond to exogenous stimuli and react accordingly in a “collective manner” (Fuseler and Valarmathi, 2012), somewhat similar to the cooperative migration of mesenchymal cells during differential tissue development during embryogenesis, and may be during cancer invasion and metastasis (Davies, 2013). The responses of interest with respect to stem cell biology and tissue engineering include cellular migration to a specific location and cessation of migration when the target region has been reached and thereby control of cellular proliferation.

Live-cell time-lapse microscopy provides a way to track the dynamics of single-cell response. In addition, it enables one to obtain a high-resolution dynamic picture of the timing of various responses. Single-cell information is often very important, since there can be a considerable degree of stochastic cell-to-cell variability in a response, as a result of, for example, subtle variations in the number of downstream signaling molecules or organelle such as ribosomes in each cell or its exact cell-cycle state at any given moment (Lim et al., 2015). Thus, when one looks at a large population of cells or a collective response of a group of cells (Fuseler and Valarmathi, 2012), critical information about the nature (spatial and temporal) of a signaling response could be lost. Time-lapse microscopy of live cells provides one to overcome these limitations. On the whole, time-lapse microscopy provides us a powerful way to follow single-cell response, and is the best way to follow the dynamics of these responses, considering the fact that MSCs are fundamentally a heterogeneous population of cells.

In a wound healing model of cellular migration, directed movement of postnatal MSCs into the non-cellular regions of the wound zone is significantly suppressed and inhibited by the presence of exogenous NO. The suppressive effects on cellular migration, proliferation, and morphological changes in postnatal MSCs are proportional to the time of exposure of NO. The longer the time of exposure of postnatal MSCs to NO, the more pronounced are the suppressive effects. This is readily seen in the time-displacement response curves, comparing control MSCs and MSCs treated with NO for a short or a long period of time.

### Janus effect of NO on cellular migration of normal and stem cells

Suppression of cellular migration by NO is similar in adult cells obtained from various tissues, including endothelial cells (Smolenski et al., 2000), vascular smooth muscle cells (Sarkar et al., 1996; Kiviluoto et al., 2001; Seymour et al., 2010), epithelial cells, and keratinocytes (Lindsay et al., 2007). The effect of NO on cellular migration of stems cells is highly variable, further revealing the Janus nature of NO on cellular functions. Consistent with our observations on adult MSCs, the proliferation of multipotent MVSCs is inhibited by increased NO in a dose dependent manner (Curtis et al., 2014). However, in contrast to our previous observation on adult MSCs (Fuseler and Valarmathi, 2012) in which NO appears to enhance the onset of differentiation of MSCs, the further differentiation of MVSCs to mesenchymal-like stem cells was inhibited by NO (Curtis et al., 2014). Suppression of NO has alternate effects on different types of stem cells. Suppression of NO production in bone marrow-derived EPCs significantly reduced cellular migration, proliferation, and angiogenesis of tube formation (Lu et al., 2015), indicating that these various cellular functions require NO (Lindsay et al., 2007). However, suppression of NO promotes the directed migration of pluripotent stem cell-derived neural stem cells toward cancer target cells, improving stem cell tumor tropism (Chen et al., 2013). These observations would indicate that NO does not have a generalized inhibitory or stimulatory effect on stem cells. The effect of NO is highly variable and appears to be specific for each stem cell phenotype. In the broadest terms, NO appears to be a mediator of cellular movement in both normal and stem cells, and functions mainly to slow or stop directed cellular migration.

The effect of exogenous NO on MSC's directed migration is directly time dependent. The longer the cells are exposed to NO, the greater is the suppression of directed cellular migration. The total distance MSCs migrated into the wound area is progressively diminished by continuous exposure to NO. Long term exposure of MSCs to NO in a static monolayer for 7 days (group SN-2) prior to migration resulted in maximal reduction in the total length of the migration path as well-direct migration into the wound area. Lateral movement of MSCs is slightly higher in the presence of NO (SN-1 and SN-2 groups), but is not significantly different from the lateral movements seen in the control MSCs. This observation suggests that molecular elements comprising both the mechanical mechanisms of motility and the directionally of motility are altered by NO in static cells prior to movement, and in cells that are just initiating movement. The greatest effect is seen in MSCs exposed to long term NO. The potential mechanism for this suppression of movement could occur by nitrosylation of specific proteins that are directly involved in the control of actual cellular movement and directed signaling.

The extent of movement into the wound area, or cellular displacement, can be controlled by the time of exposure of the MSCs to NO. In the controls, MSCs displacement into the wound area continuously increases over the 24 h time period. A typical displacement curve and its regression analysis curve for control MSCs are illustrated in Figure 5A. In general, the application of NO immediately following the formation of the wound area results in very different displacement curve. Here the initial movement of the MSCs is slow for the initial 4 h, then movement rapidly increases for between 6 and 8 h after which directed cellular movement ceases or expresses more random movement. Displacement of MSCs in group SN-2 exhibits little directed movement into the wound area for the initial 10 h. These MSCs then undergo slow directed migration for between 8 and 10 h, after which directed migration ceases. These findings indicate that the effects of NO are additive, with the suppression of movement greater when the MSCs are exposed to NO for a longer period of time. The displacement into the wound area of the control MSCs and MSCs exposed to NO for different times can be elegantly described by a four parameter sigmoid non-linear expression [*y* = *y*_*o*_ + *a*/(1+exp(–(*x*–*x*_*o*_)/*b*))], where *x* is time in hours and *y* is the displacement distance in μm. In this expression, the coefficient ***a***, which is associated with the distance component of the expression decreases with increasing exposure to NO. This would suggest that the motive components responsible for cell movement are suppressed by NO in a time-dependent manner. The two most logical potential targets for this effect of NO may include the actin cytoskeleton responsible for cellular movement and the production of super oxide radicals, which are a requirement for actin-mediated cellular movement. The mechanism of action of NO, could be by the nitrosylation of actin (Clancy et al., 1995; Aslan et al., 2003), which suppresses the polymerization of G actin to F actin. Limiting the availability of G-actin subunits to form F-actin would slow the recycling of the subunits, slowing the treadmilling of the actin filaments that are responsible for directed movement. Another interesting mechanism may involve the nitrosylation of the elements of the RhoA/Rock pathway (Nour-Eldine et al., 2016) or other components of NADPH-Oxidase family, which could inhibit the assembly of the NAD(P)H complex resulting in the inhibition of MSCs movement. Interestingly, the coefficient ***b***, which is associated with the time component of the function, is decreased to the same level regardless of the time of exposure to NO. This would indicate that the effect of NO on the timing of movement is not cumulative and is rapidly established in both the SN-1 and SN-2 groups.

### NO alters MSCs mean migration velocity

The regulation of cellular movement by NO is further demonstrated by its effect on the velocity of MSCs engaged in directed movement. The presence of exogenous NO significantly suppresses the mean velocity of migrating MSCs. MSCs exposed to NO in the SN-1 and SN-2 groups express the same reduction in migration velocity. This would suggest that there is no cumulative effect of NO on cell migration velocity. The longer time exposure of MSCs to NO does not induce a greater reduction in cellular velocity. The suppressive effect of NO on cellular velocity must act immediately on cells that are in the process of migration. This would suggest the presence of a specific target critical for controlling cell movement which is only available to the effect of NO when the MSCs are actively moving. This target could be associated with the production of super oxide radicals required for actin-mediated cell movement, or with the actual force producing components of the cytoskeleton required for movement or regulation of these components.

The suppressive effect of NO also extends to cell division of MSCs. In the presence of NO, cell division is inhibited in both the SN-1 and SN-2 groups. This would suggest that NO may have direct effect on the ability of the cell to form a functional mitotic or meiotic spindles (Gelaude et al., 2015), may suppress actin mediated cytokinesis or enter cell division cycle (Phung et al., 2006; Pandey et al., 2010; Pandey and Chaube, 2015). These observations suggest that NO may more specifically affect cytoskeletal proteins that are associated with cell division. Increased NO has been shown to result in loss of microtubules in malignant cells (Laguinge et al., 2004). Studies have suggested that S-nitrosylation can impact proteins that are associated with cell division, such as the microtubule-associated protein 4 (Greco et al., 2006), tubulin α-6 chain, tubulin α-4 chain, and actin 2/7 (Lindermayr et al., 2005). Bearing in mind that the functionality and morphogenesis of the mitotic or meiotic spindle are a self-organized process, depending on microtubule dynamic, microtubule associated proteins, and motor proteins interactions. Any disorganization of these elements by S-nitrosylation or nitro-tyrosine formation can lead to spindle dysfunction and suppression of cellular proliferation (Eiserich et al., 1999; Phung et al., 2006). Further, studies are required and are in progress, specifically to identify which one of these components are affected by NO leading to suppression of MSCs proliferation.

### Fractal dimension (*D*) of MSCs

Concurrent with suppression of the migration and division of MSCs, NO alters their cellular morphology. In the presence of NO, the migrating MSCs encounter a significant reduction in cellular area that is in accordance with the NO induced reduction of cellular area observed in other types of mesenchymal cells (Lindsay et al., 2007). In contrast to other cell types, in the presence of NO, MSCs do not round-up its morphology but become smaller and retain its stellate to polygonal type of cellular morphology (Fuseler and Valarmathi, 2012). The morphological changes induced by NO in MSCs are variable, but consistent patterns are revealed by examination of the fractal dimension (*D*) of the control MSCs and MSCs migrating in the presence of NO. In control MSCs, the initial 2–4 h of migration are characterized by a decrease in (*D*) values. This suggest that early migration of individual cells is characterized by more uniform and directed cellular movement. The cells would be expressing fewer random cytoplasmic projections and those projection produced are directionally organized and orientated. As MSCs migrate into the more open space of the wound area, their (*D*) value increases slightly to maintain a relative constant steady state value with variable oscillation over the 24 h time period.

In MSCs exposed to NO immediately following wound formation (group SN-1), the change in cellular (*D*) value is opposite that seen in the controls. The initial (*D*) values for the two groups are the same, however, in the SN-1 group the (*D*) significantly increases between 2 and 6 h before declining to a relatively steady state value. The steady value for (*D*) of the SN-1 group is greater than that of the control group. This indicates that the presence of NO results in a greater level of chaos associated with the production of cytoplasmic process associated with directed migration. This continued flux and disorganization of the cellular membrane persist even after the MSCs have ceased their migration. The cell membrane continues producing filopodia and lamellipodia, but exhibits no movement.

Continuous exposure to NO has the most dramatic effect on MSCs' morphology expressed as their (*D*) value. The initial (*D*) for the MSCs in the SN-2 group is greater than that of both the SN-1 group and controls. These MSCs exhibit a modest decrease in (*D*) when the cells initiated directed migration and are moving into the wound area between 2 and 6 h. With suppression of cell migration at 10 h, the (*D*) value remains elevated and essentially attained the steady state value. The MSCs of the SN-2 group exhibit extensive production of cytoplasmic protrusions and filopodia in random directions when directed movement has ceased. This extensive production of randomly produced cytoplasmic protrusion would account for the increased (*D*) value, and increased cytoplasmic chaos associated with these cells. NO appears to be able to suppress directed movement of MSCs, but does not completely inhibit the molecular machinery used for cell movement. The continued excessive activity of the plasma membrane would suggest that these cells are capable of movement, but the signal of in which direction to move is not received or cannot be acted upon.

In all three groups, the initiation of cellular movement is characterized by an initial decrease in (*D*), which then increases to a steady state value as the cells are migrating. This would indicate the conversion of MSCs at rest to those that are capable of migration evolve as well-increase in order of the elements responsible for cellular shape and movement, i.e., the actin cytoskeleton. Once the cells begin to move, a certain degree of disorder is associated with migration as seen by the increase in (*D*) to a steady state value. These changes in (*D*) during cellular migration may very well-reflect the continuous changes in the actin cytoskeleton associated with cellular movement.

In MSCs migration, as shown in this study, the presence of NO appears to act as a stop signal for individual cell movement. NO can also stop the collective migration of MSCs but does not suppress their activity or function, as shown in our previous study (Fuseler and Valarmathi, 2012). The dual capacity of NO, i.e., the presence of NO to suppress MSCs movement and the absence of NO to promote movement would be advantageous in embryonic development and in bioengineered tissue reconstructs. These suppositions suggest that movement of MSCs and other stem cells may be regulated in a redox dependent manner or by reversible nitrosylation. Both of these options in control of cellular movements are attractive and may actually be interactive. Expression of ROS appears to modulate the activity of actin binding and actin regulatory proteins leading to the formation of membrane ruffles and integrin mediated cell spreading (Nimnual et al., 2003). Facilitation of ROS production at the site of injury in wound healing assay contributed to migration toward the injury site (Ikeda et al., 2005). In addition, cytoskeletal proteins, including actin (Neumann et al., 2006), associated with actin-mediated motility have been found to express altered functions as a result of nitration (Dalle-Donne et al., 2000). Reversible S-nitrosylation of proteins forming S-nitrosothiols (SNO) associated with motility is an attractive alternate (Clement, 2014; Hlaing and Clement, 2014). Actin has been demonstrated to possess SNO sensitive sites (Su et al., 2013) suggesting the importance of redox regulation in the formation G to F-actin cycling. Nitrosylation of actin suppresses its ability to polymerize (Lu et al., 2009). Such conditions that are decreasing the amount of F-actin would suppress actin mediated cellular movement and related functions. Exogenous NO has been demonstrated to suppress ROS production by S-nitrosylation of the Nox subunits of NAD(P)H oxidase (Qian et al., 2012). Reduction of ROS would result in suppression or reduction of cellular movement.

NO suppresses cell division. This may occur when NO affects the mitotic spindle formation or by suppressing the cytokinesis. NO may suppress cell division by interfering with actin-mediated cleavage furrow formation that separates the two daughter cells. These cells may undergo endomitotic nuclear division without cytoplasmic division (cytokinesis). Further, experiments would be needed to resolve this observation.

## Conclusions

Here, we speculate that NO may suppress or alter the migration patterns of individual adult tissue-specific stem cells, especially marrow-derived postnatal MSCs, by modulating the status of regulatory proteins associated with actin-mediated movement, or by modulating the functionality of the cytoskeletal proteins involved in motility. Thus, migration of individual MSCs appears to be regulated by the small free-radical gas molecule, NO, by providing a slowdown or stop signal. NO also apparently suppresses mitosis of migrating MSCs. By having the capacity to stop stem cells at a specific location either in embryonic development or in bioengineered constructs, NO may also act as an early differentiation-inducing agent for MSCs, and priming them for further phenotypic differentiation as presented in our previous study (Fuseler and Valarmathi, 2012).

## Author contributions

JF: Conception and design, administrative support, collection and assembly of data, data analysis and interpretation, manuscript writing, final approval of manuscript. MV: Conception and design, financial support, provision of study material, collection and assembly of data, data analysis and interpretation, manuscript writing, final approval of manuscript. JF and MV contributed equally to this work.

## Funding

“This work was supported by an award from the American Heart Association.”—National Scientist Development Grant (11SDG5280022) for MV.

### Conflict of interest statement

The authors declare that the research was conducted in the absence of any commercial or financial relationships that could be construed as a potential conflict of interest.

## Supplementary material

Video 1 **Valarmathi—Video—1—MSCs—Control-24 H-10X Ph.AVI**. https://doi.org/10.6084/m9.figshare.4012425.v1

Video 2 **Valarmathi—Video—2—MSCs-200 μM SNAP-SN-1-24 H-10X Ph.AVI**. https://doi.org/10.6084/m9.figshare.4012431.v1

Video 3 **Valarmathi—Video—3—MSCs-200 μM SNAP-SN-2-24 H-10X Ph.AVI**. https://doi.org/10.6084/m9.figshare.4012428.v1
